# Supplementary material for: PRECIS-2 used as an implementation science tool for global environmental health: A cross-sectional evaluation of the Ecolectivos study protocol to reduce burning of household plastic waste in rural Guatemala
Source: PLoS One. 2024 Dec 30;19(12):e0316161. doi: 10.1371/journal.pone.0316161 (PMC11684596; doi:10.1371/journal.pone.0316161)
Supplement: Attachment — Submitted filename: RESPONSE TO REVIEWERS_2024Oct3.docx [file pone.0316161.s002.docx]

RESPONSE TO REVIEWERS:

1) Please double-check grammar (e.g. repetition and verb tense)

Reviewer Response: Thank you we have we have reviewed and modified the grammar throughout the text.

2) Please double-check refs (e.g. punctuation and formatting)
   a. The refs. need to be refined and integrated more thoroughly throughout the text, as there are several statements without citations. For example, more information on the validity, reliability, subjectivity, or generalisation, especially re, the proposed RCT—could be strengthened with additional references (e.g. 10.1186/s13063-023-07313-0; 10.1016/j.jclinepi.2020.03.027).

Reviewer Response: We have fixed the references and added references where they are needed throughout the text.

**ABSTRACT REVIEWER COMMENTS**

3) The Abstract is clear and well-structured, but certain refinements are needed:
   a. First, the transition between terms, specifically the connection between RCTs and the PRECIS-2 tool, is abrupt. The link between the efficacy of the tool and its application could be more clearly outlined. A simple addition explaining how the tool applies to RCTs and enhances their design in real-world contexts can resolve this.

Reviewer Response: We were constrained by the guidelines not to exceed 300 words but have tried to address (3 a-g) all of which pertain to the abstract, to the best of our ability. The abstract is now 450 words.

We have re-written the first few sentences to clarify the relationship between efficacy and effectiveness and the function PRECIS plays in the evaluation of pragmatism in trial designs. We hope this resolves the reviewer’s concerns.

b. For example, the debate on "RCTs being underutilised" would be more suitable as an in-depth part of the Introduction rather than the Abstract.

Reviewer Response: We feel that is an important part of the argument, that RCTS are underutilized in environmental health studies, and thus should be included that in the abstract, but we elaborated more on this in the introduction as the reviewer suggested.

c. If the authors prefer to elaborate on RCTs to provide a stronger rationale, consider making a clear link between RCTs and the intervention. If the intention is to argue against other, this argument could be reframed within the Introduction.

Reviewer Response: We are not sure if we interpreted the reviewer’s comment correctly, but the purpose of the PRECIS-2 is to evaluate the degree of pragmatism of an RCT. We used this tool to evaluate our protocol that focuses on a randomized intervention delivered in a community setting to address an environmental health concern. We tried to clarify that more in the abstract. We are not clear about the second sentence and what “other” refers to.

d. Please clarify the real-world application of the tool, including its background and the framework supporting its use.

Reviewer Response: We have clarified this in lines 3-6 of the abstract.

e. The Methods section could begin with an overview of the overall characteristics, followed by an introduction to the PRECIS-2 framework. This will refine readability while also adhering to the guidelines.

Reviewer Response: We moved the sample and study characteristics to the beginning of the methods section and then described the PRECIS-2 tool.

f. Some parts of the Results section need explanation re. their importance to the findings.

Reviewer Response: We have strengthened the importance of the findings in lines 30-33.

g. Please work on a stronger conclusion debating the applications and implications for environmental health, for example-- I understand that the debate on RCTs at the beginning of Introduction was provided in similar studies (e.g. 10.1136/bmj.h2147), but it may not be appropriate for a broader audience in this current form.

Reviewer Response: We have rewritten the conclusion.

4) The authors report the ID (i.e. NCT05130632) but provided the STROBE checklist, also mentioned as observational. Although this isn't wrong, some concerns were noted:
   a. Neither the STROBE nor CONSORT guidelines appear to be adhered to in the ms. The - important - items based on the study design could be addressed to ensure compliance. I suggest a thorough check of the applicable guidelines (i.e. STROBE or CONSORT) and a careful revision of both the Abstract and the ms.

Reviewer Response: We included the STROBE guidelines to describe this cross-sectional (observational) study. We have reviewed the criteria and clarified in the manuscript. Some are N/A (e.g. flow diagram). We provided the NCT trial number because this cross-sectional study is evaluating the protocol for a randomized controlled trial. The NCT provides more detail about the study that is being evaluated. We also include a brief overview of the trial in the introduction to introduce the reader to the study. For that reason, we only include the STROBE guidelines only for the present manuscript.

b. Insufficient detail regarding the sample, design, and background, in both Abstract and text, making it unclear what the variables, outcomes, and implications are.

Reviewer response: We have added details about the sample, design and background to the abstract as described above in 1). We have also added details about sample and design in the methods as described in 5). We have elaborated on the Background as described in 4c). We hope that this is sufficient to address these concerns.

c. In its conciseness, the Background section describes the intentions. There are interesting explanations re. the importance of RCTs over other designs (e.g. "This is especially relevant in community-based environmental studies"). This construction of argument is important for setting the tone for both the readers and a broader audience. However, despite the commendable attention to detail, the authors appear to be aware of the limitations in clearly defining their study. In the Study Details section, the context, rationale, and gaps are somewhat overextended (e.g. "Household air pollution from solid fuel combustion is a major environmental risk factor in low- and middle-income countries, accounting for an estimated 2.6 million deaths annually (World Health Organization, 2018). The contribution of plastic waste incineration in household fires has not been quantified. This is problematic for countries like Guatemala, where 71% of households burn waste as a primary means of disposal (Government of the Republic of Guatemala, 2019)."). While informative, this introduction of the problem is not as clear as it could be. I highly suggest reshaping the Introduction to improve flow and readability, focusing on: a) background, b) rationale, c) main gaps in previous studies, d) the motivation behind this study, e) its significance, and f) the hypotheses and objectives.

Reviewer response: Thank you for highlighting our intended use of the PRECIS-2 to evaluate a protocol for a community-based intervention study to address an environmental problem. We reorganized the introduction as suggested, explicitly stating “rationale” “gaps” “motivation” significance” and “objectives”. We are not testing hypotheses in this exploratory study. We removed the statement about the lack of global waste management in the introduction but left it in the discussion, to contextualize the comments made by the evaluators who reviewed the protocol using the PRECIS-2 tool. We have looked throughout the paper for the statements (e.g. "Household air pollution from solid fuel combustion is a major environmental risk factor in low- and middle-income countries, accounting for an estimated 2.6 million deaths annually (World Health Organization, 2018). The contribution of plastic waste incineration in household fires has not been quantified. This is problematic for countries like Guatemala, where 71% of households burn waste as a primary means of disposal (Government of the Republic of Guatemala, 2019).") but realize that these statements refer to the actual RCT that we are conducting which is not part of the manuscript under review. Those statement are background for the larger study described in the protocol, therefore we did not address this reviewer comment.

d. Please streamline the Method section, emphasising why specific aspects or markers are important. Currently, some details are repeated from other studies rather than focusing on improving the clarity of the hypotheses and objectives.

Reviewer response: Methods have been streamlined and describe the present study. We described the sample, the sampling method, and the survey tool that was used to evaluate the protocol. We also describe the demographic variables that were collected. We hope that this improves the clarity of the objectives of this manuscript.

5) Both the Methods and Results sections lack the necessary detail, making it difficult to evaluate the manuscript in its current form. I'll list a few questions for the authors - especially based on the guidelines and necessity for refine scientific communication.
   a. Why not begin by describing a key aspect of your study—the sample?

Reviewer response: We have added a description of our intended sample at the beginning of the methods section and the actual sample at the beginning of the results section.

b. Where are the power calculations or sample size determinations? This is *essential*. Additionally, consider addressing the differences in the invitee ratios

Reviewer response: While we understand the importance of power analyses and sample size calculations in studies that are testing hypotheses, this was an exploratory study with 25 participants who reviewed our protocol. We have clarified that we did not conduct a power analysis in the data analysis section, lines 234-235.

We had already addressed the differences in the invitee ratios in the discussion but added a clarifying phrase in lines 211-215.

c. Please ensure adhering to the guidelines when reporting the data storage and handling

Reviewer response: We have added this verbiage to lines 182-184.

d. What are the confounding factors and possible intervening variables, and how were they managed? Consider response bias as a primary issue

Reviewer response: We wish that we had a larger sample size to examine potential demographic covariates, but with 25 participants we could not. We did instead attempt to examine differences based on external evaluators versus Ecolectivos investigators, with the caveat that there would be potential bias as elaborated upon in the results (lines 486-488).

e. Please include details on the psychometric properties of the tool, including the scoring and rating process

Reviewer response: The PRECIS-2 tool has not been evaluated as rigorously as one might when developing scales to measure latent variables for psychological instruments (e.g., stress, anxiety) that use underlying measurement theory. In (f) we describe the validity and reliability testing that has been undertaken by the developers of the instrument.

f. Was the tool validated?

Reviewer response: Yes, the PRECIS-2 tool has been validated, and we have emphasized that in several areas and explained the validation in lines 178-180.

g. Why is no mention of the specific tests, their assumptions, parameters and explanations? Although Mann-Whitney is mentioned, no explanation is provided. Please consider detailing all parameters, including effect sizes and Cis

Reviewer response: In lines 176-179 of our original draft we stated “Variable distributions were evaluated to assess whether assumptions of normality were met; nonparametric tests were used based on these findings. We used the Mann Whitney-U Test to compare medians across the two groups (Ecolectivos investigators compared with external evaluators).” We rewrote the section to try to make it more clear about why we chose the Mann-Whitney test. We did not estimate an effect size in this cross-sectional study because we are not conducting hypothesis testing or estimating a treatment effect. We did provide confidence intervals since we know that our small sample does not represent a given population and are not attempting to estimate a measure of central tendency and our confidence around that measure if the survey were to be repeated.

h. A detailed section for the qualitative part it essential

Reviewer response: We revised the qualitative section and provided references to describe the thematic analysis of the short responses that were provided in our online survey. See lines 202-208

i. Explain the modifications to the study

Reviewer response: The modifications are the results of an NIH-funded supplement that we received to add environmental health promotors and a dissemination component to the control group at the end of the study. Because this was not evaluated in the protocol for this study, and would be too confusing to explain, we removed it.

6) There seems to be a misunderstanding, as the Results section includes aspects that should belong in the Methods section.

Reviewer response: We have moved the two questions that were asked from the results section to the methods section (Lines 163-166). We have removed the scoring criteria descriptions; they are now in the methods section. We believe that is what the reviewer is referring to when stating that methods are in the results section.

a. More detail on eligibility, analyses, and scoring methods are relevant.

Reviewer response: We have modified the methods section to address these concerns.

b. How the data were collected or measured, details of survey questions, and the scales used for evaluation could be provided in Methods.

Reviewer response: We have modified the methods section to address these concerns.

c. Correlations are also advisable - as well as potential regression analyses.

Reviewer response: Each of the domains are independent so we do not feel that correlations would be warranted, and these have not been reported in the literature with PRECIS-2 in other publications as far as we can determine. With only 25 participants, we feel that we do not have the statistical power to do a regression analysis, nor do we have a single outcome, instead we have 9 outcomes, thus we are underpowered to uses regression techniques.

7) The formative phase baseline recruitment, involving door-to-door approaches, was considered more explanatory due to the additional effort required, while recruitment for the main trial was viewed as pragmatic, with village-level randomisation and community-based approaches that aligned with usual community engagement. Please elaborate.

Reviewer response: We have re-written the section to clarify the statement.

8) The authors should consider checking their data again. For example, in Tables:
   a. In Table 2, there are no descriptions of additional tests (e.g. chi-squared). Also check of how these influence the other variables.

Reviewer response: Given the small sample size of this exploratory study, we did not calculate X2 tests between Ecolectivos members and external evaluators since the cell sizes were even smaller (with some cells being = 0) than what is presented in Table 2 which presents an overall view of the 25 participants. We did not assess whether age, gender, race, ethnicity influenced how external evaluators versus Ecolectivos members selected scores in each of the 9 domains based on the rational describe in (6c) above.

b. In Table 3, please expand including effects, CIs and check outliers.

Reviewer response: Given the small sample size of this exploratory study, we did not conduct regression analyses to check the effects of demographic variables. Since we did not conduct inferential statistical analyses, we did not calculate CIs. We did not estimate an effect size in this cross-sectional study because we are not assessing a treatment effect.

c. Considering the small sample, the IQRs can be double-checked

Reviewer response: Two investigators re-ran the data and we found two numbers that were rounded to whole numbers, representing the Likert scale, which we corrected. We infer that the reviewer is puzzled by the 25^th^ or 75^th^ percentile being the same as the 50^th^ percentile, but that is indeed the case in which several of these domains-- if everyone ranked similarly, the 50^th^ and the 25^th^ or the 75^th^ might be the same.

d. In Table 4, the first two domains exhibited significant results after correcting the data. Even without adjusting, the results for recruitment were also significant. I'd highly suggest the authors to double-check.

Reviewer response: We re-ran the data and the results are correct as presented. We did not correct the data, nor did we adjust the data for other covariates. There is no overlap in the eligibility data when looking at the boxplots, with a slight overlap for the recruitment data (data not shown).

9) There is the need to expand a few more demographics, specifically the ones that could serve as confounding factors as IQ, education or previous experiences.

Reviewer response: We did not collect data on IQ. All were college educated (PhD or MD) and recruited from academic settings (which we clarified in the methods).

10) The dispersion of data (i.e. IQR) indicates both the possibility of influence of pre-existing knowledge, as that sample could be biased, i.e. if sample were larger, then probably outcomes would be different. This can be better observed.

Reviewer response: We assume this is in reference to Table 3. We are not surprised that the external evaluators would score higher than our team members since they were purposely chosen for their intermediate to expert knowledge of implementation science. Given the small sample size of this exploratory study, we did not detect a statistically significant difference.
